# SHANK3 Co-ordinately Regulates Autophagy and Apoptosis in Myocardial Infarction

**DOI:** 10.3389/fphys.2020.01082

**Published:** 2020-08-25

**Authors:** Wanrong Man, Jing Gu, Bo Wang, Mingming Zhang, Jianqiang Hu, Jie Lin, Dong Sun, Zhenyu Xiong, Xiaoming Gu, Kaikai Hao, Baolin Guo, Gaoli Wei, Liang Zhang, Rui Song, Congye Li, Haichang Wang, Dongdong Sun

**Affiliations:** ^1^Department of Cardiology, Xijing Hospital, Fourth Military Medical University, Xi’an, China; ^2^School of Basic Medicine, Fourth Military Medical University, Xi’an, China; ^3^Department of Cardiology, Tangdu Hospital, Fourth Military Medical University, Xi’an, China; ^4^Department of Physiology and Pathophysiology, Fourth Military Medical University, Xi’an, China; ^5^Heart Hospital, Xi’an International Medical Center, Xi’an, China

**Keywords:** SH3 and multiple ankyrin repeat domains 3, myocardial infarction, autophagy, apoptosis, cardiomyocytes

## Abstract

Cardiac remodeling and dysfunction are responsible for the high mortality after myocardial infarction (MI). We assessed the potential for Shank3 to alleviate the post-infarction cardiac dysfunction. The experimental MI mice model was constructed by left anterior descending coronary artery ligation. Shank3 knockout aggravated cardiac dysfunction after MI, while Shank3 overexpression alleviated it. The histological examination showed that the infarct size was significantly increased in the acute phase of MI in the Shank3 knockout group, and the cardiac dysfunction of the Shank3 knockout group was even more severe than the Shank3 overexpression group, revealed by echocardiography analyses. *In vitro*, cultured neonatal cardiomyocytes were subjected to simulated MI. Shank3 downregulation curbed LC3 expression and autophagosome-lysosome fusion. Furthermore, Shank3 downregulation increased cardiomyocyte apoptosis. In contrast, Shank3 upregulation induced autophagy, and inhibited apoptosis under hypoxia. *In vivo*, western blot analysis showed decreased levels of Atg7, Beclin1, LC3-II, and Bcl-2 as well as increased expression of p62, cleaved caspase-3, and cleaved caspase-9 in the Shank3 knockout group which suffered from MI. On the other hand, it also revealed that Shank3 overexpression induced autophagy and inhibited apoptosis after MI. Shank3 may serve as a new target for improving cardiac function after MI by inducing autophagy while inhibiting apoptosis.

## Introduction

Globally, myocardial infarction (MI) remains a big challenge (Likosky et al., 2018). Cardiac remodeling and progressive heart failure after MI have been a major cause of mortality (Arnold et al., 2014; Bucholz et al., 2016). Although many interventions have been reported to alleviate cardiac remodeling and dysfunction in animal models, the vast majority of them has proved clinically less effective against MI injury. Cardiac remodeling after MI is a complex process involving various mechanisms contributing to cardiac structural and functional injury (Prabhu and Frangogiannis, 2016; Gibb and Hill, 2018). Autophagy is a physiological and regulated process in which a cell removes unnecessary or dysfunctional components via the lysosomal degradative pathway (Shirakabe et al., 2016a; Del Re et al., 2019; Wu et al., 2019). In response to MI, increased levels of autophagy can also promote cell survival (Shirakabe et al., 2016b; Sciarretta et al., 2018a). Uncontrolled apoptosis can worsen cardiac function after ischemia and hypoxia (Frangogiannis, 2015). Hence, the regulation of autophagy and apoptosis is essential for the restoration of cardiac function.

Shank proteins are post-synaptic scaffold proteins, which are crucial for proper synaptic development and function (Monteiro and Feng, 2017). The Shank3 gene is located on mouse chromosome 15E3 (human: 22q13.3) and spans 60 kilobases of genomic DNA (Monteiro and Feng, 2017). SHANK3 (or ProSAP2) regulates the synaptic formation, development, and plasticity (Mossa et al., 2018). Phelan–McDermid syndrome (PMS) symptoms such as autistic-like behaviors, hypotonia and delayed or absent speech are due to SHANK3 haploinsufficiency (Monteiro and Feng, 2017). The expression of Shank3 vary in different organs of rodents. It is highly expressed in the brain, heart and spleen (Lim et al., 1999). Shank3 has been reported to regulate autophagy, apoptosis, proliferation, and development (Grasselli et al., 2020). However, the role of Shank3 in post-infarction cardiac remodeling is not well understood. In this study, we constructed Shank3 knockout and transgenic mice to recapitulate MI pathogenesis and assessed the restoration of cardiac function after MI.

Our study revealed a novel molecular, Shank3, participated in MI pathology and identified Shank3 as a potential therapeutic target against MI injury.

## Materials and Methods

### Genetically Modified Mice

Shank3 knockout (Shank3^–/–^) and Shank3 transgenic (Tg-Shank3) mice were generated on the C57BL/6 background by K&D gene technology (Wuhan, China). Animals were genotyped by real-time PCR. Age-matched male mice (6–8 weeks, 20–25 *g*) were used in all animal experiments. The experimenters were blind to group assignment and outcome assessment. All mice procedures were performed in compliance with the Guide for the Care and Use of Laboratory Animals and the Guidelines for the Welfare of Experimental Animals issued by Ethics Committee on Animal Care of the Fourth Military Medical University.

The mice were randomly allocated into the following groups with *n* = 30 each: (1) wild-type (WT); (2) Shank3^–/–^; (3) MI; (4) MI + Shank3^–/–^; (5) non-transgenic mice (NTg); (6) Tg-Shank3; (7) MI + NTg; and (8) MI + Tg-Shank3.

### Myocardial Infarction Surgical Procedures

The surgical procedures were carried out under anesthesia which was induced by 2% isoflurane inhalation. A left thoracotomy was performed at the level of the fifth intercostal space to expose the heart. The left anterior descending branch (LAD) of the coronary artery 2–3 mm from its origin was ligated permanently by a knot made around with a 6–0 silk suture. The heart was rapidly pushed back into the chest, and then the muscles were reclosed. The type of skin sutures was 5–0 nylon. Sham-operated animals received the same surgical procedures without LAD ligation. The mice recovered in a cage with the temperature maintained at 37°C under humane care. The survival status of each group after surgery was shown in the Supplementary Material. Myocardial death was evaluated by 2,3,5-triphenyltetrazolium chloride (TTC) staining (*n* = 4–7/group). For detailed procedures, please refer to the previous description (Gao et al., 2010; Valentin et al., 2016).

### Echocardiography

After 28 days of MI, M-mode Echocardiography was conducted with the aid of an echocardiography system with a 15-MHz linear transducer (Visual Sonics Vevo 2100, Toronto, ON, Canada) (*n* = 4–8/group). Left ventricular end-diastolic diameter (LVEDD) and left ventricular end-systolic diameter (LVESD) were measured. Left ventricular ejection fraction (LVEF) and left ventricular fraction shortening (LVFS) were calculated by computer algorithms.

### Immunoblotting

The following antibodies were used: Shank3 (#ab93607, Abcam), Beclin1 (#ab62472, Abcam), p62 (#ab91526, Abcam), cleaved caspase-3 (#ab2302, Abcam), cleaved caspase-9 (#a0281, ABclonal), Bax (#a19684, ABclonal), Bcl-2 (#a19693, Abclonal), LC3A/B (#12741S, CST), Atg7 (#67341-1-Ig, Proteintech); and the secondary antibodies (anti-mouse/rabbit IgG) conjugated with horseradish peroxidase. Details of the immunoblotting procedure were described previously (Hu et al., 2016).

### Primary Neonatal Cardiomyocytes Culture

Primary cultures of cardiomyocytes were prepared from 1-day-old non-transgenic mice. Cells were cultured in DMEM supplemented with 10% fetal bovine serum (Hyclone SV30087.02, Logan, UT, United States) and 1% penicillin/streptomycin and maintained at 37°C in 5% CO^2^ as previously described (Hu et al., 2016).

### Construction and Transduction of Adenoviruses

Adenovirus encoding HBAD-mRFP-GFP-LC3 was purchased from Hanbio Technology Ltd. (Shanghai, China). Adenoviruses expressing a short hairpin (sh) RNA directed against Shank3 (Ad-sh-Shank3), and harboring Shank3 (Ad-Shank3) and control vectors (Ad-LacZ, Ad-sh-LacZ) were purchased from Hanbio Technology Ltd. (Shanghai, China). The titers of adenoviruses were 1 × 10^10^ PFU/ml. The multiplicity of infection used was 80:1. The shRNA sequence targeting mouse Shank3 was GACCACTGATGAGAATGGTTGGCAA.

Adenoviruses about Shank3 (Ad-sh-Shank3, Ad-Shank3, Ad-LacZ, and Ad-sh-LacZ) were transduced 24 h after the transduction of HBAD-mRFP-GFP-LC3. After 24 h, the cardiomyocytes were treated with hypoxia for 8 h as previously described (Hu et al., 2016).

### Detection of GFP-LC3 and Aggresomes

Fluorescence detection of GFP-LC3, autophagolysosomes (APL), and autophagosomes were observed under the Olympus (Japan) FV1000 laser confocal microscope as previously described (Hu et al., 2016).

### Determination of Cardiomyocytes Apoptosis

Apoptosis was detected by the Cell Death Detection kit (Roche) after hypoxia for 8 hs as previously described (Hu et al., 2016). Olympus FV1000 laser confocal microscope was used. Apoptotic index = the number of TUNEL-positive cardiomyocytes/the total number of cardiomyocytes.

### Hypoxia

Cardiomyocytes were transferred into an ischemic buffer and simulated ischemia was performed in a humidified cell culture incubator (5% O^2^, 95% CO^2^, 37°C) for 8 h.

### Public Data and Statistical Analysis

To analyze gene expression, public repository data were employed such as BioGPS^1^, NCBI Gene Expression Omnibus (GEO) profiles^2^, and GeneNetwork (a free scientific web resource)^3^. Firstly, we visited BioGPS’s homepage and searched for the target gene. Secondly, we selected the species of the mouse. Then we read the gene report and downloaded the raw data. Finally, we used R language software to analyze the original data and export figures. R language source code was provided in the Supplementary Material.

Statistical analyses were conducted with the aid of GraphPad Prism 7 (GraphPad Software, La Jolla, CA, United States). Data visualization was done using R (version 3.3.2, The R Foundation for Statistical Computing ISBN 3-900051-07-0). Statistical analyses of differences between groups were done by Student’s *t* test, one-way ANOVA with a Fisher’s *post hoc* comparison test or two-way ANOVA with multiple *post hoc* comparisons. Significant values were defined as *p* < 0.05.

## Results

### Shank3 Alleviates Experimental MI Cardiac Injury

**FIGURE 1:**
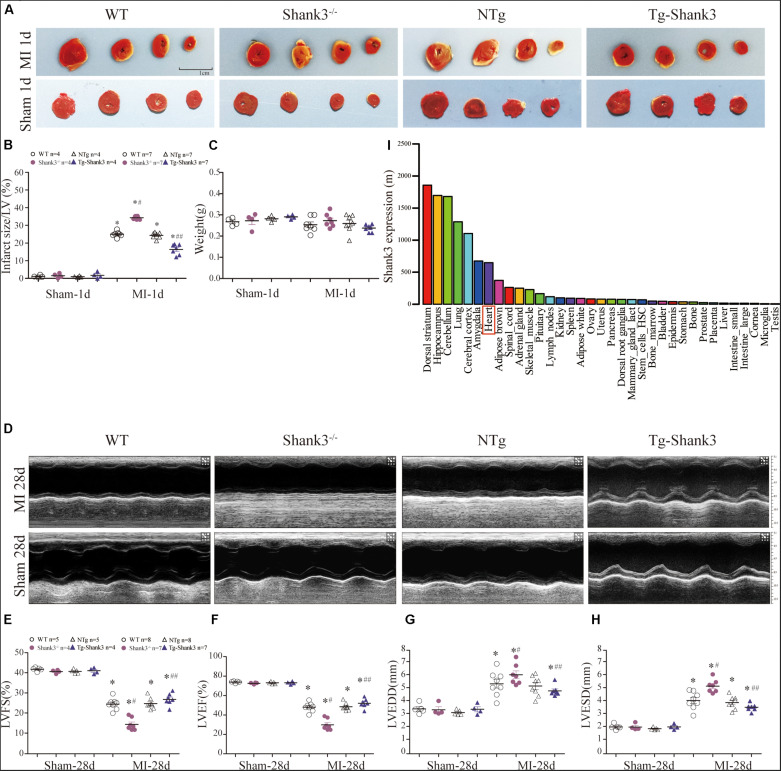
Shank3 alleviates experimental MI cardiac injury. WT, Shank3 KO, and Shank3 TG mice were subjected to sham or MI injury (ischemia 1 day or 28 days). **(A)** Representative photographs of triphenyltetrazolium chloride (TTC) staining in WT, Shank3 KO, and Shank3 TG hearts; **(B)** Quantification of TTC-stained infarct; **(C)** weight of heart; **(D)** Representative echocardiographic images at 4 weeks after MI; **(E–H)** Measurements of LVFS, LVEF, LVEDD, and LVESD; **(I)** Protein differential expression in normal mice tissues for Shank3 Gene. *p < 0.05 vs. sham-operation control; #*p* < 0.05 vs. MI + WT; ##*p* < 0.05 vs. MI + NTg (*n* = 4–8/group). Data are means and SEM. LVFS, left ventricular fraction shortening; LVEF, left ventricular ejection fraction; LVESD, left ventricular end-systolic diameter; and LVEDD, Left ventricular end-diastolic diameter.

In line with the BioGPS profiles, NCBI GEO profiles also demonstrated that Shank3 expression existed in the heart of the mouse (Figure 1I and Supplementary Material). To gain insight into the function of Shank3 in MI cardiac injury, loss-of-function and gain-of-function approaches were used in the MI mice model. We first evaluated the final MI 1-day post-MI. Histological examination demonstrated a significantly increased infarct size in Shank3 KO mice in comparison to WT group after MI, but the infarct size of Shank3 TG group was smaller than NTg group (Figures 1A–C). No infarction was observed in sham-operated groups.

Then, echocardiography analyses were employed to evaluate the heart function. Four weeks of permanent coronary ligation caused severe cardiac dysfunction as evidenced by decreased LVEF, and LVFS with increased LVEDD and LVESD (Figures 1D–H). But LVEF and LVFS were significantly lower in the MI + Shank3^–/–^ group compared with the MI group (Figures 1E,F). Shank3 knockout significantly promoted the increase in LVESD and LVEDD caused by MI (Figures 1G,H).

### Shank3 Knockout Inhibits Autophagy and Autophagosome-Lysosome Fusion

**FIGURE 2:**
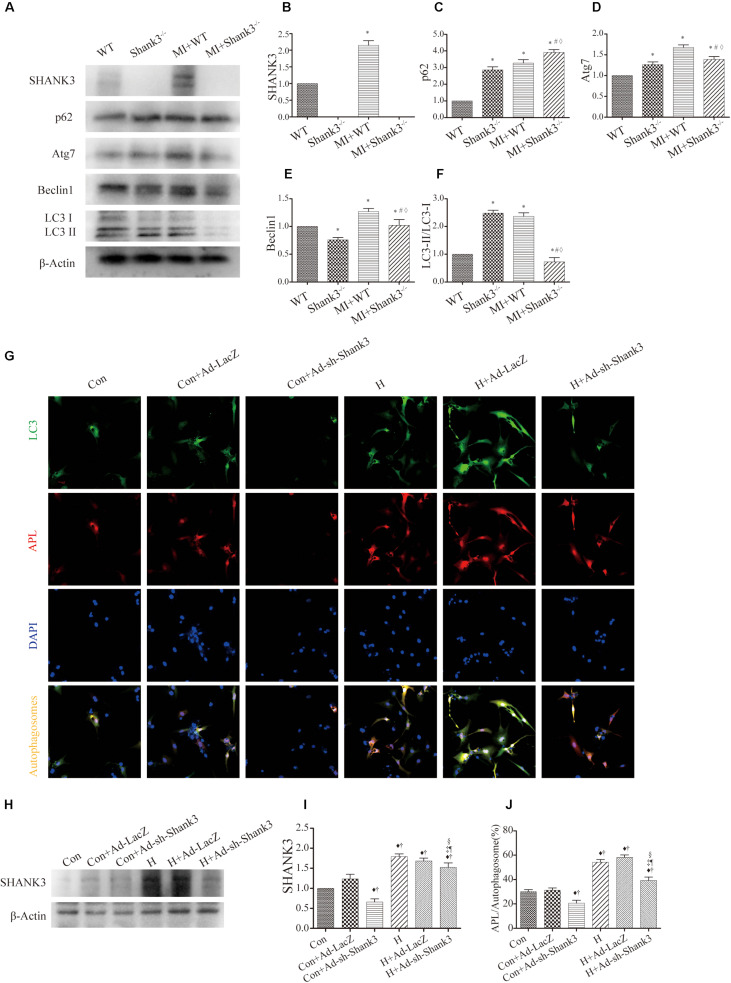
Shank3 knockout inhibits autophagy and autophagosome-lysosome fusion in cardiomyocytes. **(A–F)** Immunoblots and quantitative analyses of SHANK3, p62, Atg7, Beclin1, LC3-II/LC3-I, and β-actin *in vivo*. [*n* = 4–8/group; **(G)**] Shank3 knockout decreased the numbers of red and green puncta in cardiomyocytes transduced with Ad-GFP-mRFP-LC3 after hypoxia. Immunofluorescence staining for LC3 (green), autophagolysosomes (APL, red), DAPI (blue), and autophagosomes (orange). **(H,I)** Immunoblots and quantitative analyses of SHANK3 *in vitro*. **(J)** Quantitative analyses of APL/autophagosomes. *p < 0.05 vs. WT; #*p* < 0.05 vs. MI + WT; ♦*p* < 0.05 vs. Shank3^– /–^; ♦*p* < 0.05 vs. Con; †*p* < 0.05 vs. Con + Ad-LacZ; ‡*p* < 0.05 vs. Con + Ad-sh-Shank3; §*p* < 0.05 vs. H; ¶*p* < 0.05 vs. H + Ad-LacZ. Con, control and H, hypoxia.

Western blot analysis showed decreased Atg7, Beclin1, and LC3-II protein levels in the hypoxia group as compared with the control group. Besides, there was a significantly elevated level of p62 expression in the Shank3^–/–^ group compared with the control group (Figures 2A–F).

In cardiomyocytes transduced with HBAD-mRFP-GFP-LC3, Ad-sh-Shank3 transfection decreased green puncta number as compared with the control group under normal or hypoxia condition (Figure 2G). Under hypoxia, Ad-sh-Shank3 transfection significantly decreased autophagosome-lysosome fusion (Figures 2G–J). There was a significant increase in SHANK3 after hypoxia culture and a significant decrease after Ad-sh-Shank3 transfection as evaluated by western blotting (Figures 2H,I).

### Shank3 Knockout Induces Cardiomyocytes Apoptosis

**FIGURE 3:**
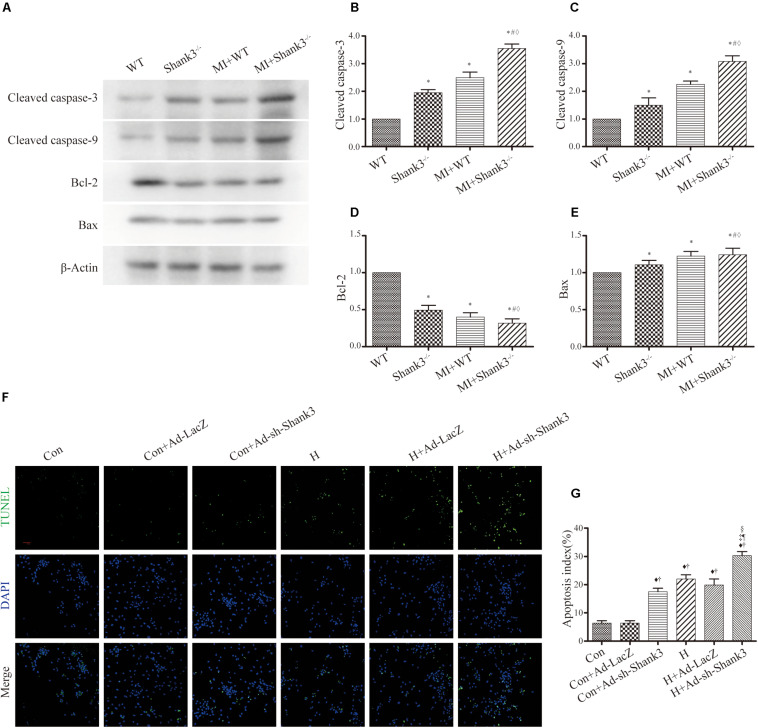
Shank3 knockout induces cardiomyocytes apoptosis. **(A–E)** Immunoblots and quantitative analyses of cleaved caspase-3, cleaved caspase-9, Bcl-2, Bax, and β-actin. (*n* = 4–8/group) **(F,G)** Representative images of TUNEL-stained primary neonatal cardiomyocytes (F) and apoptotic index (G). *p < 0.05 vs. WT; #*p* < 0.05 vs. MI + WT; ♦*p* < 0.05 vs. Shank3^– /–^; ♦*p* < 0.05 vs. Con; †*p* < 0.05 vs. Con + Ad-LacZ; ‡*p* < 0.05 vs. Con + Ad-sh-Shank3; §*p* < 0.05 vs. H; ¶*p* < 0.05 vs. H + Ad-LacZ. Con, control and H, hypoxia.

Levels of cleaved caspase-3 and cleaved caspase-9 were upregulated by Shank3 knockout in the MI + Shank3^–/–^ group (Figures 3A–C). Bcl-2 protein levels decreased after MI, but the levels of Bax showed no evident change (Figures 3A–E).

Concomitantly, photomicrograph images showed that TUNEL-positive cardiomyocytes were more abundant in the hypoxia groups than in the control groups; this effect was notable by Shank3 knockout (Figures 3F,G).

### Shank3 Overexpression Induces Autophagy

**FIGURE 4:**
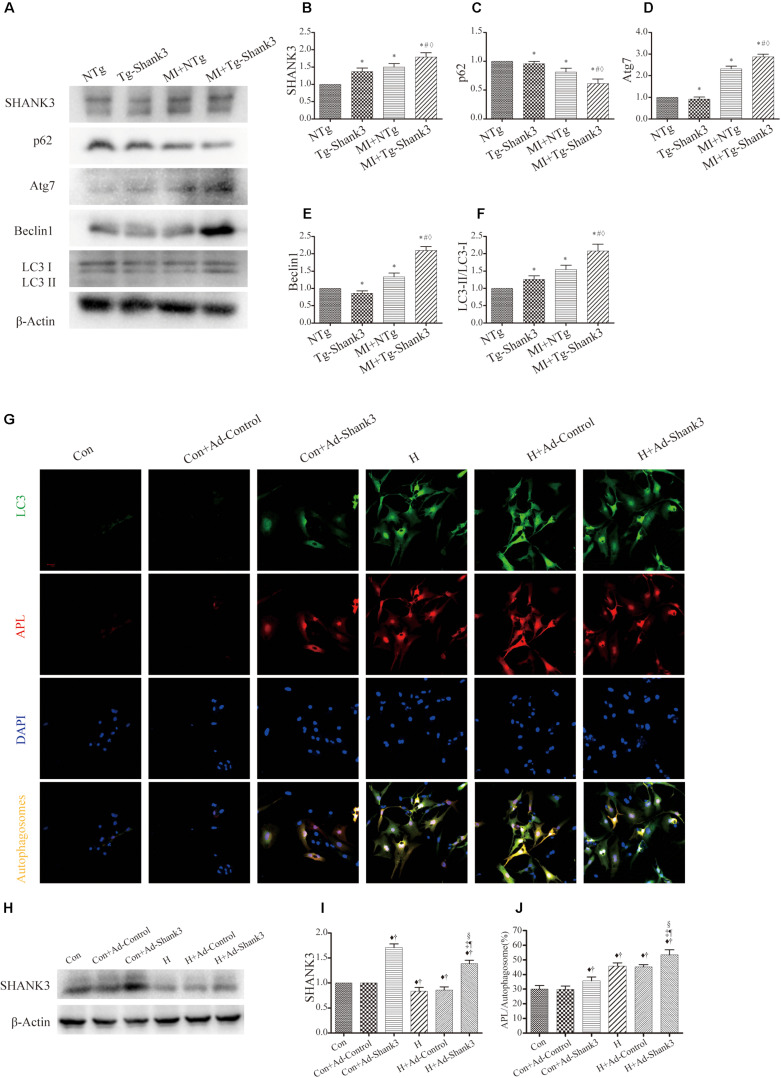
Shank3 overexpression induces autophagy and increases the accumulation of protein aggresomes in cardiomyocytes. **(A–F)** Immunoblots and quantitative analyses of SHANK3, p62, Atg7, Beclin1, and LC3-II/LC3-I and β-actin *in vivo*. (*n* = 4–8/group) **(G)** Shank3 overexpression increased the numbers of red and green puncta in cardiomyocytes transduced with Ad-GFP-mRFP-LC3 after hypoxia. Immunofluorescence staining for LC3 (green), autophagolysosomes (APL, red), DAPI (blue), and autophagosomes (orange). **(H,I)** Immunoblots and quantitative analyses of SHANK3 *in vitro*. **(J)** Quantitative analyses of APL/autophagosomes. *p < 0.05 vs. NTg; #*p* < 0.05 vs. MI + NTg; ♦*p* < 0.05 vs. Tg-Shank3; ♦*p* < 0.05 vs. Con; †*p* < 0.05 vs. Con + Ad-Control; ‡*p* < 0.05 vs. Con + Ad-Shank3; §*p* < 0.05 vs. H; ¶*p* < 0.05 vs. H + Ad-Control. Con, control and H, hypoxia.

In the hypoxia group, western blot analysis showed significantly increased Shank3, Atg7, Beclin1, and LC3-II protein levels as compared with the control group (Figures 4A–F). Meanwhile, the p62 expression of MI + Tg-Shank3 was lower than the control group (Figures 4A–C).

To investigate whether SHANK3 enhanced autophagy, we transduced the neonatal mice cardiomyocytes with adenoviruses harboring Shank3 (Ad-Shank3) and LacZ (Ad-LacZ). In cardiomyocytes transduced with HBAD-mRFP-GFP-LC3, Shank3 overexpression significantly increased the number of green puncta when compared with the control group under both normal and hypoxia conditions (Figures 4G–J). When cardiomyocytes underwent hypoxic preconditioning, the autophagosome-lysosome fusion and the level of intracellular autophagy increased significantly (Figures 4G–J).

### Shank3 Overexpression Inhibits Cardiomyocytes Apoptosis

**FIGURE 5:**
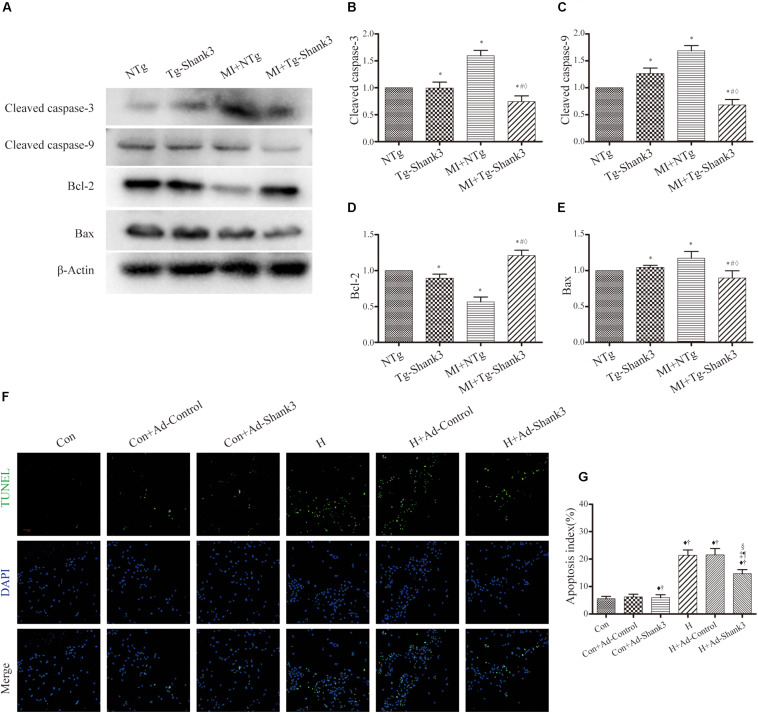
Shank3 overexpression inhibits cardiomyocytes apoptosis. **(A–E)** Immunoblots and quantitative analyses of cleaved caspase-3, cleaved caspase-9, Bcl-2, Bax, and β-actin. (*n* = 4–8/group) **(F,G)** Representative images of TUNEL-stained primary neonatal cardiomyocytes **(F)** and apoptotic index **(G)**. *p < 0.05 vs. NTg; #*p* < 0.05 vs. MI + NTg; ♦*p* < 0.05 vs. Tg-Shank3; ♦*p* < 0.05 vs. Con; †*p* < 0.05 vs. Con + Ad-Control; ‡*p* < 0.05 vs. Con + Ad-Shank3; §*p* < 0.05 vs. H; ¶*p* < 0.05 vs. H + Ad-Control. Con, control and H, hypoxia.

Protein levels of cleaved caspase-3 and cleaved caspase-9 were increased after MI injury, but western blot analysis revealed a downward trend in Shank3 overexpressing transgenic mice (Figures 5A–C). The level of Bcl-2 significantly increased in MI + Tg-Shank3 (Figures 5A–E).

Furthermore, representative TUNEL images also demonstrated that Shank3 overexpression resulted in a decrease in the ratio of apoptotic cardiomyocytes cultured under hypoxia (Figures 5F,G).

## Discussion

SHANK proteins are a family of scaffold proteins organizing the postsynaptic density of excitatory synapses and are indispensable for normal brain function. Several studies show that SHANK3 is responsible for autism spectrum disorders (ASDs), intellectual disability (ID), and schizophrenia (Durand et al., 2007; Moessner et al., 2007; Leblond et al., 2014). Recent research shows that Shank3 also plays an important role in metabolism and mitochondria function (Lee et al., 2017; Grasselli et al., 2020). *In vivo*, the metabolism of the heart and nervous system is relatively active, and both are more sensitive to hypoxia injury. We found that the expression level of Shank3 in the heart is only second to that in the nervous system through bioinformatics analysis (Figure 1I and ESM). Thus, we hypothesized that Shank3 may also play an essential role in the process of cardiac ischemia and hypoxia.

To investigate whether the Shank3 deficit affects cardiac function after MI, we used Shank3 knockout and transgenic mice to establish the experimental mouse model of MI.

Interestingly, we observed that Shank3 knockout in the experimental mouse model exacerbated the area of MI and cardiac function. However, the overexpression of Shank3 showed potential protection.

Heart failure after MI has high morbidity and mortality, and cardiac remodeling is the main cause of heart failure, but the pathological mechanism of this process is not yet clear (Arnold et al., 2014; Bucholz et al., 2016; Prabhu and Frangogiannis, 2016; Wang et al., 2016; Gibb and Hill, 2018). Previous studies have shown that the levels of autophagy and apoptosis of cardiomyocytes after MI are directly involved in the recovery of cardiac function and cardiac remodeling (Kappel et al., 2016; Sciarretta et al., 2018b; Del Re et al., 2019).

*In vitro*, a reasonable level of autophagy can protect cells by removing harmful proteins from them (Wu et al., 2019). Our previous research proved that the increase of autophagy level helped to restore cardiac function and alleviate cardiac remodeling under hypoxic injury conditions (Hu et al., 2016). On the contrary, if the level of autophagy cannot be increased correspondingly after suffering the injury, it will lead to further deterioration of heart function (Hu et al., 2016; Packer, 2020).

In our study, both levels of LC-3 and autophagosome maturation showed a significant reduction in Shank3 deficit groups after MI. On the contrary, the level of autophagy in the Shank3 overexpression group was increased to deal with waste organelles and proteins after injury. The evidence above made us believe that Shank3 had an indispensable role in the autophagy of cardiomyocytes. Meanwhile, we hypothesized that Shank3, as a scaffold protein, may provide a platform for the biological function of autophagy-related proteins (Shaw and Filbert, 2009). This viewpoint needs to be confirmed by further research.

In addition, our research also investigated cardiomyocyte apoptosis. *In vivo* or *in vitro*, the higher level of apoptosis was observed in the low expression of Shank3 groups as compared with the control groups. Several studies have shown that in the environment of nutritional deprivation and a lack of growth factors, the heart will induce autophagy and reduce apoptosis so that cardiomyocytes can survive (Del Re et al., 2019; Rabinovich-Nikitin et al., 2019; Cao et al., 2020; Davidson et al., 2020).

Our study first reports the key role Shank3 plays in functional recovery after cardiac ischemia and hypoxia by promoting autophagy and inhibiting apoptosis. Our results sheds light on a new research direction for cardiac remodeling and heart failure after MI, and Shank3 may also become a new therapeutic target for the diseases.

## Data Availability Statement

The raw data supporting the conclusions of this article will be made available by the authors, without undue reservation.

## Ethics Statement

The animal study was reviewed and approved by the Fourth Military Medical University Ethics Committee on Animal Care.

## Author Contributions

DDS and HW defined the topic of this project and revised the manuscript carefully. WM, BW, MZ, and JH carried out the laboratory experiments and wrote the manuscript. JL, DS, ZX, KH, GW, LZ, RS, and BG analyzed the data. JG translated literature and polished the manuscript. CL and XG were responsible for the animal model establishment. All authors carried out the work, read, and approved the final manuscript.

## Conflict of Interest

The authors declare that the research was conducted in the absence of any commercial or financial relationships that could be construed as a potential conflict of interest.

## Footnotes

1 http://biogps.org/

2 http://www.ncbi.nlm.nih.gov/geoprofiles

3 http://www.genenetwork.org/
