# Supplementary material for: SHANK3 Co-ordinately Regulates Autophagy and Apoptosis in Myocardial Infarction
Source: Front Physiol. 2020 Aug 25;11:1082. doi: 10.3389/fphys.2020.01082 (PMC7477314; doi:10.3389/fphys.2020.01082)
Supplement: Supplementary file 2 [file Table_2.DOCX]

Supplementary Material

# Supplemental methods

**TTC staining**

The 2,3,5-triphenyltetrazolium chloride (TTC) staining is used for calculation of infarct size in heart tissues. We use TTC as a redox indicator to differentiate between metabolically active and inactive tissues. Specifically, when TTC is applied to slides of heart tissues following induction of infarct, the viable tissue is stained red, while the non-viable infarct remains white.

Four slides (2-3 mm thick) per heart were prepared for TTC staining according to the protocol (Valentin et al., 2016). After incubating in 1% TTC (Sigma-T8877), we took pictures with a stereological microscope coupled with a camera at 10X magnification. The viable and non-viable tissue were measured via the popular open source softwßare Fiji (version: 2.0.0-rc-69/1.52i; Fiji is Just ImageJ).

**R language source code**

##barplot
##
a=read.table("GSE10246 v1.txt",header=T,sep="\t",row.names=1)
par(mar=c(6,2,2,1))
barplot(a$mean,ylim=c(0,2500))
barplot(a$mean,
 names.arg=rownames(a),
 ylim=c(0,2500),
 las=2,
 width=2,
 col=rainbow(8),
 cex.names = 0.3,
 cex.axis = 0.5, )

par(mar=c(9,6,6,4))
barplot(a$mean,ylim=c(0,2500),main="height")

p=barplot(a$mean,names.arg=rownames(a),ylim=c(0,2500),axisnames=F)
text(p,0,
 labels=rownames(a),
 srt=30,
 cex=0.4,
 adj=c(1,1),
 col="black",
 xpd = TRUE
)

#############################################
p=barplot(a$height,names.arg=rownames(a),ylim=c(0,200),axisnames=F)
text(p,0,
 labels=rownames(a),
 srt=30,
 cex=1,
 adj=c(-1,-1),
 col="blue",
 xpd = TRUE
 )

**shRNA sequence**

SiRNA1：

| Top strand | AATTCGACCACTGATGAGAATGGTTGGCAAttcaagagaTTGCCAACCATTCTCATCAGTGGTCttttttg |
| --- | --- |
| Bottom strand | GATCCAAAAAAGACCACTGATGAGAATGGTTGGCAATctcttgaaTTGCCAACCATTCTCATCAGTGGTCg |

SiRNA2：

| Top strand | AATTCGCACTACACTGTGGGTTCCTATGACAttcaagagaTGTCATAGGAACCCACAGTGTAGTGttttttg |
| --- | --- |
| Bottom strand | GATCCAAAAAACACTACACTGTGGGTTCCTATGACATctcttgaaTGTCATAGGAACCCACAGTGTAGTGCg |

SiRNA3：

| Top strand | AATTCGGGAGAAGTTGGATGAGATCCttcaagagaGGATCTCATCCAACTTCTCCCttttttg |
| --- | --- |
| Bottom strand | GATCCAAAAAAGGGAGAAGTTGGATGAGATCCTctcttgaaGGATCTCATCCAACTTCTCCCg |

SiRNA4：

| Top strand | AATTCGCAGCCTTGACTACAGCTCTGGAGAAttcaagagaTTCTCCAGAGCTGTAGTCAAGGCTGttttttg |
| --- | --- |
| Bottom strand | GATCCAAAAAACAGCCTTGACTACAGCTCTGGAGAATctcttgaaTTCTCCAGAGCTGTAGTCAAGGCTGCg |

SiRNA5:

| Top strand | AATTCGGAAGTCACCAGAGGACAAGAttcaagagaTCTTGTCCTCTGGTGACTTCCttttttg |
| --- | --- |
| Bottom strand | GATCCAAAAAAGGAAGTCACCAGAGGACAAGATctcttgaaTCTTGTCCTCTGGTGACTTCCg |

# Supplementary Figures and Tables

## Supplementary Figures


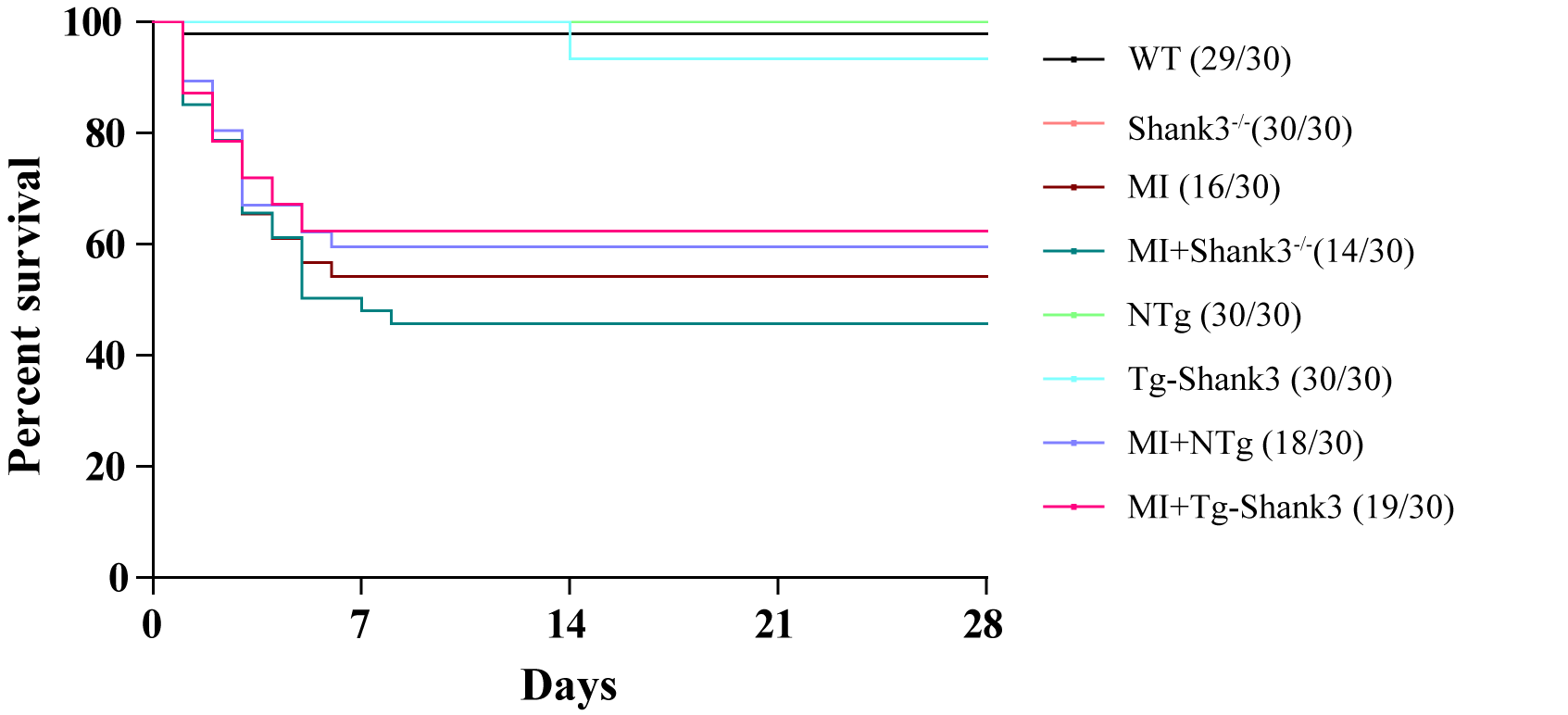


**Supplementary Figure.** Survival curves (n=30). Survival curves were analyzed by Gehan-Breslow-Wilcoxon test (Chi square=81.9 p＜0.0001).
