# Supplementary figures and images for: SHANK3 Co-ordinately Regulates Autophagy and Apoptosis in Myocardial Infarction
Source: Front Physiol. 2020 Aug 25;11:1082. doi: 10.3389/fphys.2020.01082 (PMC7477314; doi:10.3389/fphys.2020.01082)

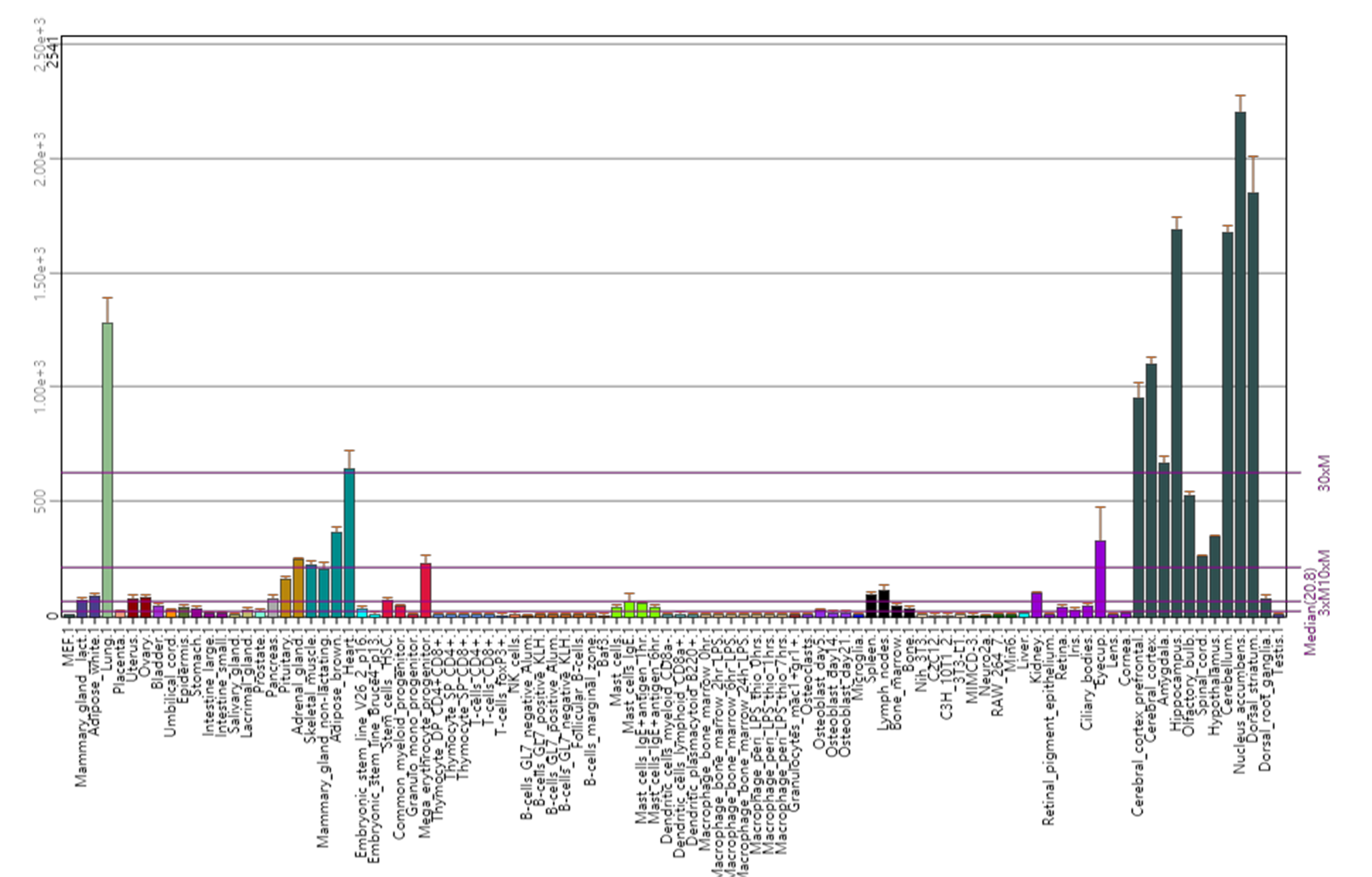

Supplement: Supplementary file 4 [file Image_1.TIF]
